# Differential Expression of Motilin Receptor in Various Parts of Gastrointestinal Tract in Dogs

**DOI:** 10.1155/2015/970940

**Published:** 2015-03-31

**Authors:** Yu He, Hui Wang, DongYan Yang, ChengYan Wang, LanLan Yang, Chunxiang Jin

**Affiliations:** ^1^Third Clinical College of Norman Bethune Medical Division, Jilin University, Changchun, Jilin 130033, China; ^2^Second Clinical College of Norman Bethune Medical Division, Jilin University, Changchun, Jilin 130041, China

## Abstract

*Objectives*. The presence of motilin receptor in the GI tract of different animal species has been verified. However, the quantitation of motilin receptor expression in different regions of the GI tract remains unclear. The aim of this study was to investigate the expression of motilin receptor in the GI tract and semiquantitatively compare the expression difference in different GI regions in dogs. *Methods*. Antrum, duodenum, jejunum, ileum, proximal colon, middle colon, and distal colon were obtained from various parts of the GI tract of six sacrificed dogs. The distribution of motilin receptor was determined by immunohistochemistry. The expression levels of motilin receptor mRNA in different regions were measured by RT-PCR. *Results*. Motilin receptor was expressed throughout the GI tract in dogs. Multiple comparisons of the mean motilin receptor mRNA expression among various regions were significant (*P* < 0.05). Motilin receptor mRNA was extensively expressed in duodenum, followed by ileum, jejunum, proximal colon, antrum, middle colon, and distal colon. Immunohistochemistry revealed that motilin receptor immunoreactivity was observed only in the enteric nervous system. *Conclusion*. Motilin receptor is expressed differentially along the GI tract in dogs. The significantly high expression of motilin receptor mRNA is found in the duodenum.

## 1. Introduction

Motilin is a 22-amino-acid peptide synthesized by endocrine cells of the duodenojejunal mucosa that plays a critical role in the regulation of the gastrointestinal (GI) interdigestive motility [1, 2]. It is accepted that its binding and activation of motilin receptor induce the phase III contraction of migrating motor complex (MMC) [3], which contributes to the mechanical and chemical cleansing of the empty stomach in preparation for the next meal. Thus motilin receptor has also acted as a clinical pathological target for GI motility disorder. To date, motilin receptor has been found in the human, tree shrew, dog, cow, pika, and rabbit, but not in rodents [4]. Only a motilin receptor pseudogene has been identified in rodents, which is associated with evolution of specialized rodent gastric physiology, involving loss of ability to vomit [4, 5]. Motilin is functional in mammals capable of vomiting. The exception is rabbit, the only other mammal unable to vomit, in which motilin might be conserved to regulate caecotrophy, another specialized digestive process [6, 7]. Therefore, these marked species differences exist in anatomy and controlling mechanisms, perhaps arising during evolution as a result of different diets and living environments [8]. Together, functions and expression of motilin receptor in the GI tract of numerous animal species have been verified in previous studies [2, 9–11]; however, the detailed distribution difference along the GI tract remains unclear. Undoubtedly, the knowledge of the regional expression of motilin receptor will contribute to the understanding of the effect of motilin on the GI motility. So far, most of the important findings concerning the physiology of motilin have been obtained in dogs due to the physiological similarities between the dog and human GI tract [12–15]. Therefore, in this study, we use the dog as an animal model to semiquantitatively determine the distribution and expression difference of motilin receptor along the GI tract.

## 2. Materials and Methods

### 2.1. Animals

This study was carried out in strict accordance with the recommendations in the Guide for the Care and Use of Laboratory Animals of the National Institutes of Health. The protocol was approved by the Institutional Animal Care and Use Committee of Jilin University. This study was approved by the Animal Care Committee of the Third Hospital of Jilin University. All efforts were made to minimize the discomfort. A total of 6 dogs of either sex (weighing 14–16 kg) were used, and all were purpose-bred, mixed-breed dogs.

### 2.2. Tissue Preparation

Antrum, duodenum, jejunum, ileum, proximal colon, middle colon, and distal colon were collected from 6 dogs under general anesthesia. Then, dogs were euthanatized. After being washed in physiological saline solution, all tissues were equally divided into two groups. One group was put into formalin (10%) for staining; the other group was snap-frozen on dry ice and stored at −80°C before mRNA extraction.

### 2.3. Methods

#### 2.3.1. Immunohistochemistry for Motilin Receptor Distribution in Dog GI Tract

Formalin-fixed, paraffin-embedded sections, cut at a thickness of 4 *μ*m, were deparaffinized, rehydrated, and boiled in retrieval solution. After being blocked in normal goat serum for 30 min to prevent nonspecific binding, sections were incubated with rabbit anti-dog motilin receptor antibody (1 : 200, courtesy of RaQualia, Taketoyo, Japan) overnight at 4°C. Next day, after being washed in PBS, sections were incubated for 30 min at room temperature with biotinylated goat anti-rabbit antibody and horse radish peroxidase conjugated avidin. Stained sections were visualized by DAB (Sigma, St. Louis, MO, USA). Motilin receptor immunoreactivity was shown brown on cytoplasm. Positive staining was analyzed by using Image-Pro Plus (IPP) 6.0 software. Negative control, where the primary antibody was substituted with PBS, was routinely performed.

#### 2.3.2. RT-PCR for Motilin Receptor mRNA Determination in Dog GI Tract

Total RNA from different dog tissues was extracted using Trizol reagent (Invitrogen Life Technologies, Carlsbad, CA, USA). Single-strand cDNA was synthesized from 1 *μ*g of total RNA of each tissue using two-step RT-PCR Kit (Takara, Shiga, Japan). An aliquot of cDNA was used as a template for subsequent PCR. PCR was performed to amplify the dog motilin receptor using the two primers 5′-ACCACCGCCTACTTCTTCCT-3′ and 5′-GCCTGTTTCCCTACACACCT-3′ [10] under the following condition: denaturation at 94°C (30 s); annealing at 56°C (30 s); and extension at 72°C (30 s) for 40 cycles with 10 *μ*L 2X Taq PCR MasterMix (Takara, Japan). A final extension period of 7 min at 72°C completed the PCR. The housekeeping gene for glyceraldehyde-3-phosphate dehydrogenase (GAPDH) was amplified as an internal control using the following two primers [10]: 5′-CCATCACCATCTTCCAGGAG-3′ and 5′-CCTGCTTCACCACCTTCTTG-3′. PCR products were separated on a 1.5% agarose gel (Invitrogen, CA, USA) and visualized by ethidium bromide staining. The expression of mRNA was standardized by GAPDH and demonstrated by Quantity one 4.6.2 (USA).

#### 2.3.3. Statistics

Results were shown as mean ± S.E. Statistical data analyses were performed by one-way analysis of variance (ANOVA). Multiple comparisons were assessed by least-significant difference (LSD). If a *P* value was found to be less than 0.05, then the result would be considered statistically significant.

## 3. Results

### 3.1. Immunohistochemical Distribution of Motilin Receptor in the Dog GI Tract

**Figure 1:**
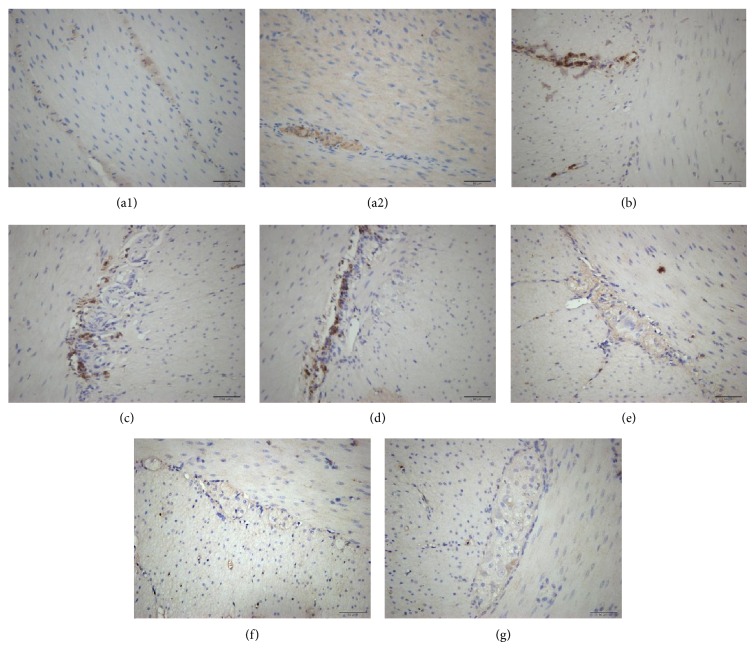
Immunohistochemical distribution of motilin receptor in the dog GI tract. Demonstration of the distribution of motilin receptor in nerve fibers among smooth muscles from the dog antrum (a1) and neuronal cell bodies in the myenteric plexus from the dog antrum, duodenum, jejunum, ileum, proximal colon, middle colon, and distal colon (×400) (a2–g). Scale bar, 50 *μ*m.

Nerve fibers among smooth muscles and neuronal cell bodies in the myenteric plexus expressing motilin receptor immunoreactivity were observed in antrum, duodenum, jejunum, ileum, proximal colon, middle colon, and distal colon of dogs. No motilin receptor immunoreactivity was found in smooth muscle cells. The duodenum and ileum were strongly immunoreactive and motilin receptor immunoreactivity weakened gradually in the more distal part of GI tract (Figure 1).

### 3.2. Differential Expression of Motilin Receptor mRNA in the Dog GI Tract

**Figure 2:**
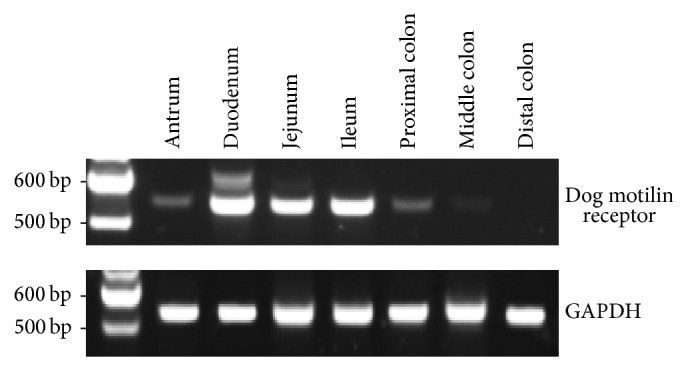
Differential expression of motilin receptor mRNA in the dog GI tract by RT-PCR. The 549 bp PCR product corresponds to the motilin receptor.

A PCR product of a predicted size of 549 bp was amplified from cDNA isolated from different regions. No PCR product was detected in the distal colon. The expressions of motilin receptor mRNA along the GI tract (antrum, duodenum, jejunum, ileum, proximal colon, and middle colon) were 0.49 ± 0.04, 1.02 ± 0.08, 0.74 ± 0.06, 0.92 ± 0.07, 0.61 ± 0.05, and 0.25 ± 0.02, respectively. Multiple comparisons of the mean motilin receptor mRNA expression among these regions were significant (*P* < 0.05, one-way ANOVA test) (Figure 2).

**Figure 3:**
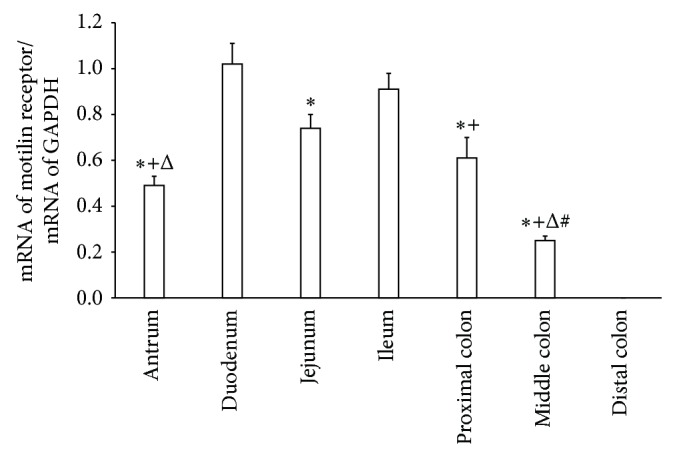
Comparisons of the mean motilin receptor mRNA expression in the dog GI tract by densitometry analysis of the bands. Intensities for the band in these regions were compared between each other. ^∗^ *P* < 0.01, compared with the band in duodenum; ^+^ *P* < 0.01, compared with the band in ileum; ^Δ^ *P* < 0.01, compared with the band in jejunum; ^#^ *P* < 0.01, compared with the band in proximal colon or antrum.

Comparisons of mRNA expression in duodenum versus antrum, jejunum, and colons were significant (*P* < 0.01, LSD). There were no significant differences in mRNA expression between duodenum and ileum. Comparisons of mRNA expression in ileum versus antrum and colons were significant (*P* < 0.01, LSD). There were no significant differences between ileum and jejunum. Comparisons of mRNA expression in jejunum versus antrum, middle colon were significant (*P* < 0.01, LSD). There were no significant differences between jejunum and proximal colon. Also, there were no significant differences between proximal colon and antrum. But comparisons of mRNA expression in proximal colon and antrum versus middle colon were both significant (*P* < 0.01, LSD) (Figure 3).

**Figure 4:**
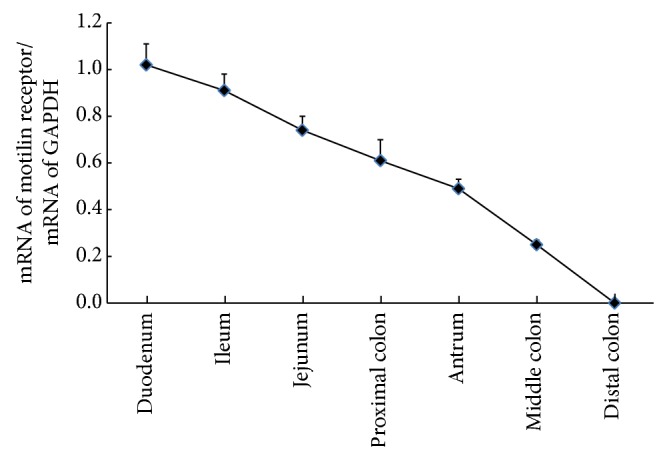
The tendency line of motilin receptor mRNA expression in the dog GI tract.

Therefore, motilin receptor mRNA was extensively expressed in duodenum. The expression concentration in the dog GI tract decreased gradually as follows: duodenum, ileum, jejunum, proximal colon, antrum, middle colon, and distal colon (Figure 4).

## 4. Discussion

Although the distribution and expression of motilin receptor along the GI tract have been debated in the past, this study reveals significant and detailed information about this topic. Our immunohistochemistry data showed the motilin receptor was only expressed in the enteric nervous system of the dog GI tract, which was in agreement with the previous study [10]. Furthermore, we found the expression levels of motilin receptor mRNA among various parts of the GI tract were different. This discovery provides the structural basis on which properties and hypothesis of motilin's action on the GI motility could be interpreted.

It has been reported that, in rabbits and humans, motilin receptors were distributed on both the myenteric plexus and the smooth muscle cells of the GI tract [2, 11, 16]. In vitro, motilin induced contraction of smooth muscle preparations isolated from rabbit and human GI tract [17]. In contrast, motilin did not stimulate contractions of smooth muscle preparations from the dog GI tract [18], which indicated that motilin's action on dogs was not through a direct interaction between motilin and smooth muscles. Our findings that no motilin receptor was found in smooth muscle cells also supported this result. However, another study showed motilin stimulated contractions in isolated perfused dog stomach that had been completely denervated extrinsically [19] and hence indicated that motilin-induced gastric contraction was mediated by the myenteric cholinergic neural pathway. This was consistent with our data that motilin receptors were exclusively localized in myenteric nervous system.

In this study, we found that the expression levels of the motilin receptor mRNA in different organs (antrum, duodenum, jejunum, ileum, or colon) were different. Our study showed the significantly high expression of motilin receptor mRNA was found in the duodenum, followed by ileum, jejunum, proximal colon, antrum, middle colon, and distal colon. This expression difference could be closely associated with the function of motilin receptor. Motilin has been recognized as an important endogenous regulator of gastrointestinal MMC, mediated by motilin receptor. So far, the mechanism of gastrointestinal MMC still remains unclear. It was hypothesized that gastrointestinal MMC was mediated by a positive feedback mechanism in the duodenum via the interaction between motilin and 5-hydroxytryptamine (5-HT) [20]. Possible mechanism was that the increased luminal pressure of duodenum resulted in 5-HT release from enterochromaffin cells (EC), which stimulated duodenal contractions and resulted in further increase of duodenal pressure, leading to duodenal MMC phase III. Finally, maximally increased duodenal pressure initiated motilin release. Released motilin in turn stimulated 5-HT release which acted on 5-HT_3_ receptors of vagal afferent, inducing gastric MMC phase III via vagovagal reflex [20, 21]. Moreover, it was confirmed that 5-HT concentration of duodenum, but not the stomach, was increased during gastric MMC phase II and phase III [20], which suggested that the positive feedback between motilin and 5-HT occurred in the duodenum. After duodenum resection, gastric MMC-like activity was initially absent but reappeared after 1–4 months as if the jejunum were the duodenum [22]. These studies pointed out the critical role of duodenum in gastrointestinal MMC and our results that maximal concentration of motilin receptor mRNA was found in the duodenum provide the structural evidence for this function.

This current study also found that motilin receptor distribution along the more distal GI tract showed a gradually decreasing gradient in density, similar to human motilin receptors [9, 16]. It is known that the usefulness of motilides (such as erythromycin) has been demonstrated for determination of dysmotility states of the human upper GI tract, ileum, and colon [17, 23–26], whereas it has no effects on distal colonic motility [27]. These results could be interpreted by the low expression of motilin receptor in the lower GI tract. Additionally, many immunocytochemical studies indicated that motilin-immunopositive cells (Mo cells) were known to exist in the upper small intestine of many species [28–34] and generally decrease in the aboral direction, which might suggest potential differences in distribution of motilin receptor and following motilin action on different regions of the GI tract.

Interestingly, the localization of motilin receptor and the region for high expression of motilin receptor are various across different species. In contrast with our results, the highest concentration of human motilin receptors was found in the antrum [9], while the rabbit motilin receptors were present in very high concentrations in the colon [2]. It is accepted that the physiological similarities of motilin are found between the dog and human GI tract. During the interdigestive state, endogenous peak plasma motilin coincided with the appearance of phase III of the MMC in the gastroduodenum of dogs and humans [12]. Immunoneutralization of circulating motilin suppressed phase III motor activity [13]; infusion of motilin at concentrations that mimic circulating levels induced premature phase III activity in both dogs and humans [14, 15]. However, our findings together with previous studies [10, 35] confirmed marked differences in the localization and the expression of motilin receptor between dogs and humans, although data showed that motilin receptors were located on smooth muscle cells as well as on enteric nerves in the rabbit and human. It was thought that motilin receptors from nerves or smooth muscles expressed different binding characteristics and pharmacological features, which confirmed the existence of 2 subtypes of motilin receptors located on nerves (N) and muscles (M) [2]. And functional studies in humans or in rabbits suggested that the neural receptor was more sensitive than the muscle receptor to stimulation by erythromycin [9, 36]. Moreover Sanger et al. [37] pointed out that motilin preferentially operated by facilitating enteric cholinergic activity rather than directly contracting the muscle, which is the exclusive acting way of motilin in dogs. It was known that the highest concentration of human motilin receptors was found in the antrum, predominantly in the neural preparation [9]. Intravenous motilin administration induced the phase III contractions in the human antrum, which were blocked by the muscarinic antagonist atropine [38]. MMC is a cyclic, recurring motility pattern that occurs in the stomach and small bowel during fasting; it is interrupted by feeding. Phase III is the most active of MMC, with a burst of contractions originating from the antrum or duodenum and migrating distally. Phase III of the MMC with an antral origin can be induced in humans through intravenous administration of motilin [39]. In contrast, in dogs, it is obvious that duodenal phase III is frequently antecedent to gastric phase III [40–43]. It is unclear whether the region for high expression of motilin receptor could be relevant to the region for MMC origin or not. But these observations suggest that the distribution and function of motilin receptors in the GI tract are variable and the biological significance of motilin receptor across species remains debatable, which might have a deep impact on translation to human physiology and pharmacology.

In this study, we proved that motilin receptors were expressed in dog myenteric system differentially but we did not determine which cell types expressed the motilin receptor. Because of the positive feedback in the duodenum, Takahashi speculated that motilin receptors could be likely located at EC cells of the duodenal mucosa, which mediate gastric MMC phase III [21]. However, Xu et al. detected the motilin receptor on membrane of interstitial cells of Cajal (ICC) of the rabbit through triple-labeled immunofluorescent staining [44], which bridged motilin with ICC and developed the mechanism of gastric motility. Whether motilin receptor could be present on ICC or EC of dogs remains to be studied to uncover possible species difference. Now, the signal transduction between the released motilin from the duodenal mucosa and the released 5-HT from the duodenal EC cells remains obscure. But it is clear that the duodenum plays an important role in initiating gastric MMC of dogs [20], which is just the place where we found the high expression of motilin receptors.

## 5. Conclusion

We found that motilin receptor was expressed differentially in myenteric nervous system throughout the dog GI tract and the significantly high expression of motilin receptor mRNA was found in the duodenum. These findings suggest that the expression difference of motilin receptor could exert different effects on various regions, which is helpful for exploring the mechanism of gastrointestinal MMC and for optimizing the treatments of patients with motility disorders.
